# Bluetongue virus outer-capsid protein VP2 expressed in *Nicotiana benthamiana* raises neutralising antibodies and a protective immune response in IFNAR ^−/−^ mice

**DOI:** 10.1016/j.jvacx.2019.100026

**Published:** 2019-06-22

**Authors:** Petra C. Fay, Houssam Attoui, Carrie Batten, Fauziah Mohd Jaafar, George P. Lomonossoff, Janet M. Daly, Peter P.C. Mertens

**Affiliations:** a The Pirbright Institute, Ash Road, Pirbright, Surrey GU24 0NF, UK; b School of Veterinary Medicine and Science, University of Nottingham, Sutton Bonington Campus, Leicestershire LE12 5RD, UK; c UMR VIROLOGIE 1161, INRA, Ecole Nationale Vétérinaire d’Alfort, ANSES, Université Paris-Est, Maisons-Alfort F-94700, France; d Department of Biological Chemistry, John Innes Centre, Norwich NR4 7UH, UK

**Keywords:** Bluetongue, BTV, Bluetongue virus, Neutralising antibodies, Subunit protein, VP2, Vaccine, Vaccination, IFNAR ^−/−^

## Abstract

Bluetongue is a severe, economically important disease of ruminants that is widely distributed in tropical and temperate regions around the world. It is associated with major production losses, restrictions of animal movements and trade, as well as costs associated with developing and implementing effective surveillance and control measures. Mammalian hosts infected with bluetongue virus (BTV) generate a protective neutralising antibody response targeting the major BTV outer-capsid protein and serotype-specific antigen, VP2. BTV VP2 proteins that have been expressed in plants are soluble, with a native conformation displaying neutralising epitopes and can assemble with other BTV structural proteins to form virus-like particles (VLPs).

His-tagged VP2 proteins of BTV serotypes 4 and 8 were transiently expressed in *Nicotiana benthamiana* then purified by immobilised metal affinity chromatography (IMAC). Antisera from IFNAR ^−/−^ mice prime/boost vaccinated with the purified proteins, were shown to contain VP2-specific antibodies by Indirect ELISA (I-ELISA), western blotting and serum neutralisation tests (SNT). Vaccinated mice, subsequently challenged with either the homologous or heterologous BTV serotype, developed viraemia by day 3 post-infection. However, no clinical signs were observed in mice challenged with the homologous serotype (either prime-boost or single-shot vaccinated), all of which survived for the duration of the study. In contrast, all of the vaccinated mice challenged with a heterologous serotype, died, showing no evidence of cross-protection or suppression of viraemia, as detected by real-time RT-qPCR or virus isolation. The induction of protective, serotype-specific neutralising antibodies in IFNAR ^−/−^ mice, indicates potential for the use of plant-expressed BTV VP2s as subunit vaccine components, or as a basis for serotype-specific serological assays.

## Highlights

• Neutralising antibodies after vaccination of mice with bluetongue VP2 proteins.
• Protection in mice against bluetongue 4 or 8, after vaccination with VP2 proteins.
• High level expression of native VP2 proteins of bluetongue 4 or 8, in tobacco plants.

## 1 Introduction

Bluetongue virus (BTV) infects a wide range of domesticated and wild ruminants, causing the severe clinical disease ‘bluetongue’ (BT) primarily in naïve sheep and some deer species [1], [2], [3]. Bluetongue outbreaks in non-endemic regions of the world, as well as those caused by exotic BTV strains/serotypes in endemic regions, can cause major economic losses. These include fatalities (mainly among sheep), loss of productivity and reproductive performance (in both cattle and sheep), restrictions of animal movement and trade [4], [5], as well as the costs of vaccination programmes and surveillance measures, which are required to demonstrate eradication before trade restrictions can be lifted [5], [6].

*Bluetongue virus* is the type species of the genus *Orbivirus*, within the family *Reoviridae* [7]. The BTV genome is composed of ten linear segments of dsRNA, encoding seven structural proteins (VP1–VP7) and five non-structural proteins (NS1–NS5) [8], [9]. The outer capsid of BTV is composed of two proteins, VP2 and VP5, encoded by genome segment-2 and -5 (Seg-2 and Seg-5), respectively. VP2 mediates cell attachment during initiation of infection and elicits a protective neutralising antibody response in infected mammalian hosts, making it a target for the development of subunit vaccines [10].

Vaccines containing live attenuated BTV strains have been used in southern Europe and in endemic regions of South Africa and the USA [2], [11], [12]. Inactivated bluetongue viruses have also been used successfully as vaccines, particularly to combat the emerging BT outbreaks in Europe [2], [13], [14], [15]. Although both of these vaccine formats can be effective as a means of BT control, they are not compatible with serological assays to distinguish infected from vaccinated animals (DIVA). In addition, the Live ‘attenuated’ BTV vaccine-strains (LAVs), cause infection in the vaccinated host and can generate severe clinical disease, with a significant level of viraemia in naïve individuals of some sheep breeds/populations [13], [16]. This can lead to infection of *Culicoides* midges (the main arthropod vectors for BTV) during feeding on the vaccinated host, resulting in onward transmission of the virus [17], [18], [19]. The use of LAVs therefore contributes to the overall genetic diversity of the circulating virus pool, providing opportunities for genome-segment reassortment between vaccine and field strains. Reassortment generates progeny viruses containing novel combinations of genome segments derived from different parental strains, that can have different transmission and virulence characteristics [16], [20], [21], [22].

Recombinant BTV proteins expressed in heterologous systems can assemble into virus-like particles (VLPs) and represent candidate materials for use in subunit vaccines [23], [24]. Plant based expression systems have several advantages for production of viral antigens, including no possibility of infection, reassortment, or reversion to virulence and consequently minimal biocontainment requirements, as well as freedom from contamination with the other viral proteins, making their use compatible with DIVA assays [13], [14].

Bacterial expressed VP2 has previously been explored as a subunit vaccine component, although incorrect protein folding resulted in loss of conformational epitopes and poor solubility, reducing both protein yield and vaccine efficacy [25]. Baculovirus-expressed VP2, VP5 and VP7 [26], or virus-vectored delivery systems (e.g. Modified Vaccinia Ankara (MVA)) [27], [28], [29] have also been used to vaccinate IFNAR ^−/−^ knockout mice, although two doses of MVA were required to elicit protection against challenge with a homologous BTV serotype [28], [29]. Plant-based expression systems produce large amounts of soluble and readily purifiable proteins with relatively low maintenance costs, and are now widely used for recombinant protein expression [30], [31].

This paper describes vaccination/challenge studies showing that VP2 of BTV-4 and BTV-8 expressed in tobacco plants (*Nicotiana benthamiana*) can be used to raise neutralising antibodies and a protective response in IFNAR^−/−^ mice.

## 2 Materials and methods

### 2.1 Expression construct and plasmid design

Nucleotide sequences for Seg-2 of BTV-4 (KP821064 - virus isolate [MOR2009/09]) and BTV-8 (KP821074 - virus isolate [NET2008/03]) available from GenBank, were used to generate constructs to express VP2 in *Nicotiana benthamiana*. The gene sequences, codon optimised for plant expression, *were* synthesised by GeneArt (ThermoFisher Scientific) with a 6x His-tag at the C-terminus (to enable purification by immobilised metal affinity chromatography (IMAC)) and flanking *Age*I and *Xho*I restriction sites. These DNA constructs were cloned individually into pEAQ-*HT* expression vectors [24], [30] to generate pEAQ-*HT*-BTV4-VP2 and pEAQ-*HT*-BTV8-VP2.

### 2.2 Transient expression

pEAQ-*HT*-BTV4-VP2 and pEAQ-*HT*-BTV8-VP2 were transformed into *Agrobacterium tumefaciens* (strain LBA4404) then cultured for 48  hrs at 28 °C at 220 rpm in Luria-Bertani medium supplemented with 50µg/mL rifampicin and kanamycin. Cultures were pelleted by centrifugation at 2500*g* and re-suspended in MMA buffer (0.1 M MES, pH 5.6, 10 mM MgCl_2_ and 100 µM acetosyringone) to an OD_600nm_ of 0.4. Bacterial suspensions were incubated at room temperature for 30 min then pressure infiltrated into 3-week-old tobacco plant leaves [24].

### 2.3 Protein purification

Infiltrated plant leaves were harvested at day 8 post-infiltration, weighed and homogenised in 3x (w/v) of extraction buffer (20 mM NaH_2_PO_4_, 300 mM NaCl, 10 mM Imidazole, 1 mM DTT, pH 8, EDTA-free protease inhibitor (Roche)). Homogenates were filtered through two layers of Miracloth (Merck Millipore) then centrifuged at 14,000*g* for 15 min.

Expressed VP2 proteins were purified from the supernatant by immobilised metal affinity chromatography (IMAC) at 4 °C. Plant lysates were passed through a Ni-NTA agarose resin column (Thermo Fisher Scientific). One column volume of wash buffer (20 mM NaH_2_PO_4_, 300 mM NaCl, 20 mM Imidazole, 0.1% Triton X-100, pH 8) was added, followed by an incubation period of 1 hr. rVP2 proteins were recovered in 2 mL fractions by adding elution buffer (20 mM NaH_2_PO_4_, 300 mM NaCl, 250 mM Imidazole, pH 8). Desalting of the recovered fractions was performed using PD-10 columns (GE Healthcare).

The purified proteins were mixed 1:1 in sterile glycerol and stored in 2 mL aliquots at –20 °C. Proteins were analysed by SDS-electrophoresis on NuPAGE Bis-Tris Mini acrylamide gels (4–12% w/v). All gels were stained with InstantBlue (Expedeon).

### 2.4 IFNAR ^−/−^ mouse vaccination and challenge studies

Nine groups of six IFNAR ^−/−^ mice (a total of 54 mice) were vaccinated by intraperitoneal injection, with 5 µg per injection of rVP2 from BTV-4, or BTV-8, or phosphate-buffered saline (PBS), mixed with 100 µL Montanide ISA V50. In the first study, two groups of mice were immunised with 2 doses given 2 weeks apart (prime and boost), of rVP2 of BTV-4, two groups with rVP2 of BTV-8 and two groups with PBS (as unvaccinated controls). Two weeks after the second vaccination, each group of mice was challenged with either BTV-4 or BTV-8 (Table 1).

**Table 1:** IFNAR ^−/−^ rVP2 BTV-4 and BTV-8 vaccination and challenge regime.

Group (*n* = 6)	Vaccination		Challenge	
	Day 0 (prime)	Day 14 (boost)	Day 14	Day 28
**Study 1 – BTV-4 challenge**				
**Group 4A** rVP2-BTV-4 vaccinated Homologous BTV-4 Challenge	BTV-4	BTV-4	–	BTV-4
**Group 4B** rVP2-BTV-8 vaccinated Heterologous BTV-4 Challenge	BTV-8	BTV-8	–	BTV-4
**Group 4C** PBS vaccinated control BTV-4 challenge	PBS	PBS	–	BTV-4

**Study 1 – BTV-8 challenge**				
**Group 8A** rVP2-BTV-8 vaccinated Homologous BTV-8 Challenge	BTV-8	BTV-8	–	BTV-8
**Group 8B** rVP2-BTV-4 vaccinated Heterologous BTV-8 Challenge	BTV-4	BTV-4	–	BTV-8
**Group 8C** PBS vaccinated control BTV-8 challenge	PBS	PBS	–	BTV-8

**Study 2 – Single-dose vaccination**				
**Group 8SA** rVP2-BTV-8 vaccinated Homologous BTV-8 Challenge	BTV-8	–	BTV-8	–
**Group 8SB** rVP2-BTV-8 vaccinated Heterologous BTV-4 Challenge	BTV-8	–	BTV-4	–
**Group 8SC** PBS vaccinated control BTV-8 challenge	PBS	–	BTV-8	–

In the second study, groups of 6 mice were immunised with a single dose of rVP2 from BTV-8, or PBS, mixed with 100 µL Montanide ISA V50, then challenged 2 weeks later with BTV-8, or BTV-4 (Table 1). All of the mice were monitored daily throughout the study and clinical signs recorded (data not shown).

### 2.5 Challenge studies

Mice were challenged with a lethal dose containing 10^3^ pfu of BTV-4 [COR2004/01 E1/BHK2], or BTV-8 [FRA2006/01 E1/BHK1] by intraperitoneal injection. For study 1 (prime and boost vaccination), blood samples were collected in EDTA on day 0 (pre-vaccination), on day 28 post-vaccination (dpv)/pre-challenge, and on days 3, 5, 7 and 25 post-challenge (dpc). For study 2 (single vaccine dose), blood samples were collected on 14 dpv/(pre-challenge) and days 3, 6 and 21 post-challenge (dpc).

### 2.6 Quantitative RT-qPCR

Total viral RNA was isolated from blood samples (100 µL per sample) using the Direct-zol RNA MiniPrep Plus kit with TRI/Reagent (Zymo Research). A one-step real-time RT-qPCR was then performed using the SuperScript III Platinum qRT-PCR Kit (Invitrogen), targeting a conserved region on BTV Segment-10 [32]. BTV genome copy number/µL was determined by generating a standard curve using RNA transcribed using a T7 promoter, from a cDNA copy of BTV Segment-10. Purified Seg-10 ssRNA was quantified using the Qubit Fluorometer and Qubit RNA HS Assay Kit (Life Technologies), where viral RNA copy numbers were calculated for each reaction using the formula (Y = X/(a × 680) × 6.022 × 10^23^), where Y = dsRNA molecules/µL; X  = g/µL of dsRNA; a = viral genome length in nucleotides (BTV Seg-10 = 822 bp); 680 is the average molecular weight per nucleotide of dsRNA [33].

Genome copy number/µL was calculated from C*_T_* values using a standard and the formula: Y = aX + b, where Y = concentration (genome copy number/µL); a = slope; b = intercept and X = *C*_T_ value. The efficiency of the assay was calculated using the formula: E% = (10-^1/slope^ − 1) × 100. C*_T_* values were plotted as the dependent and BTV Seg-10 transcript RNA standards as the independent variable.

### 2.7 Serum neutralisation test (SNT)

SNTs were performed as described by Haig *et al.* [34] using Vero cells. Plates were scored for cytopathic effect (CPE) on days 5–7, with the final read used to determine neutralisation titre as the dilution of serum giving a 50% end-point.

### 2.8 Western blot

Purified rVP2 BTV-4 and BTV-8 analysed by SDS-PAGE were transferred onto a 0.45 µM nitrocellulose membrane (BioRad) at 100 mA and 4 °C for 90 min in standard transfer buffer (10% SDS, 20 mM Tris/HCl (pH.7.5) 150 mM glycine 20% isopropanol). Membranes were incubated in blocking buffer (50 mM Tris-HCl pH 7.5, 150 mM NaCl, 5% (w/v) powdered skimmed milk) for 15 min. To detect the purified rVP2 proteins, a primary detection antibody directed against Penta-His BSA-free mouse monoclonal IgG1 (Qiagen) was used. To detect VP2-specific antibodies, serum samples collected from the mouse challenge studies were used as primary antibody. For both blots, a secondary detection antibody, anti-mouse goat F (Ab) 2 fragment IgG H + L peroxidase (Beckman/Coulter), diluted 1:750 in blocking buffer was applied. Blots were developed using a chemiluminescent substrate (Bio-Rad).

### 2.9 Indirect ELISA

An Indirect ELISA (I-ELISA) was optimised using purified rVP2 of BTV-4 and BTV-8. The VP2 proteins were diluted in coating buffer (0.05 M carbonate-bicarbonate buffer, pH 9.6) at 2 µg/mL and used to coat 96-well polystyrene maxisorp ELISA plates, at 4 °C overnight. Plates were then washed 3x with 1x PBS containing 0.05% (v/v) Tween-20. Blocking buffer (5% w/v BSA in PBS) was added to each well, plates were covered and incubated at 37 °C for 1 hr, then washed 3x as previously described. Test serum (100 µL) was added, diluted in blocking buffer (1:10) and incubated for 1 hr at room temperature. Plates were then washed 3x as previously described. Secondary anti-mouse detection antibody (100 µL diluted 1:750) conjugated to horseradish peroxidase was added to each well, incubated at room temperature for 1 hr, then washed 3x. SigmaFast OPD substrate (100 µL) was added to each well, plates incubated in the dark for 15 min then absorbance measured at 450 nm. Sample OD values were normalised by subtracting the OD value of blank controls. A cut-off value for positive samples was determined as the mean of the negative control plus one standard deviation.

### 2.10 Statistical analyses

Significant differences in group neutralisation titres and real-time RT-qPCR C*_T_* values were determined using the Kruskal-Wallis test, Minitab version 17.

### 2.11 Ethics statement

All mouse studies were performed at the National Veterinary School, Maisons Alfort, France (UMR1161, INRA-ANSES-ENVA). Immunisation protocols were approved by the ethics committee at The Pirbright Institute (license number 70/6133) and ANSES-ENVA-UPEC (license number 20/12/12-25B). Virus isolates were provided by E. Breard and C. Sailleau (Bluetongue national reference laboratory, ANSES).

## 3 Results

### 3.1 Plant expression of rVP2 of BTV-4 and BTV-8

Supplementary data associated with this article can be found, in the online version, at https://doi.org/10.1016/j.jvacx.2019.100026.

His-tagged rVP2s of BTV-4 and BTV-8, expressed at high levels (∼1.5 mg/mL each) in tobacco plants and IMAC purified, were soluble suggesting that they retain a native conformation. The size and identity of the purified proteins was confirmed by SDS-PAGE and western blot analysis (Fig. S1), generating a major band migrating at the expected size of ∼110 kDa for monomeric VP2. In both cases this protein reacted with antibody against Penta-His by western blot.

**Fig. S1:** 

### 3.2 The antibody response to prime/boost rVP2 vaccination

Six groups of six IFNAR ^−/−^ mice were vaccinated (prime and boost), two groups each with purified rVP2 of BTV-4, or rVP2 of BTV-8, or 1x PBS (the control groups). These groups were subsequently challenged with BTV-4 or BTV-8 (Table 1). Mice in group 4A (vaccinated with rVP2 of BTV-4), developed a detectable level of neutralising antibodies (nAbs) against the homologous BTV-4 by 28 dpv (pre challenge) (Tables 2 and S1). However, nAbs against BTV-4 were below the sensitivity of the assay (<2), in mice from group 4B, which were vaccinated with the heterologous rVP2 of BTV-8.

**Table S1:** 

Comparable results were also obtained for mice in group 8A, which developed a slightly weaker nAb response against BTV-8 after vaccination with rVP2 of BTV-8 (Tables 2 and S1). Group 8B mice, which were vaccinated with rVP2 of BTV-4, did not generate detectable nAb levels (<2) against BTV-8 (Tables 2 and S1). No nAbs were detected against either BTV serotype in pooled sera from the control groups (groups 4C and 8C) (Tables 2 and S1). These results are consistent with a serotype-specific nAb response to the rVP2 proteins.

Antisera from mice in each of the vaccination groups were also tested post-vaccination (pre-challenge) by ELISA against the homologous rVP2 proteins of BTV-4 or BTV-8 (Table 3). Although the results for individual animals were more variable by ELISA (Table S2) than by SNT (Table S1), there was little detectable response in any of the groups to rVP2 of either serotype on day 0 (pre-vaccination). However, by day 28 pv, the mean OD values (as well as those for almost every individual animal) against rVP2 of the homologous serotype had risen, with little or no increase in the response to the rVP2 protein of the heterologous serotype, or in the control animals against either serotype (Tables 3 and S2).

**Table S2:** 

**Fig. 1:**
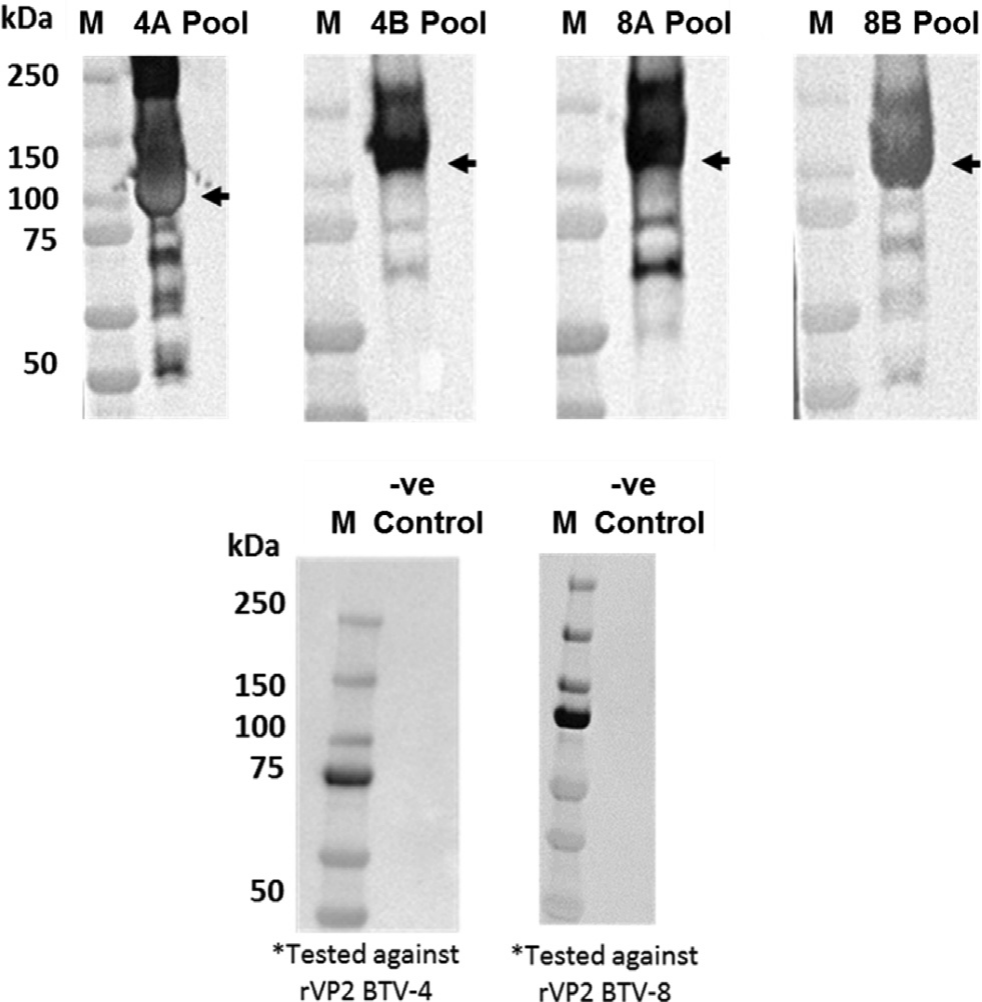
**Western blots using pooled antisera from vaccinated IFNAR ^−/−^ mice at day 28 dpv.** Pooled antisera from groups 4A and 8B were tested against rVP2 of BTV-4, while pooled antisera from groups 8A and 4B were tested against rVP2 of BTV-8. In each case bands were detected at ∼110 kDa (the MW of the VP2 monomer) as indicated by arrowheads. No equivalent bands were detected by the negative (-ve) control sera taken from groups 4C and 8C, when tested against rVP2 of BTV-4 or BTV-8 respectively.

Protein bands of the expected size for BTV rVP2 (∼110 kDa), were also detected in the purified protein preparations by western blot using the antisera taken at day 28 dpv from mice prime/boost vaccinated with rVP2 of the homologous serotype. No equivalent protein bands were detected using any of the negative control sera (Fig. 1). Due to limitations on the amount of available sera, western blots were not performed against the heterologous rVP2 proteins.

### 3.3 Challenge studies of prime/boost vaccinated mice

**Fig. 2:**
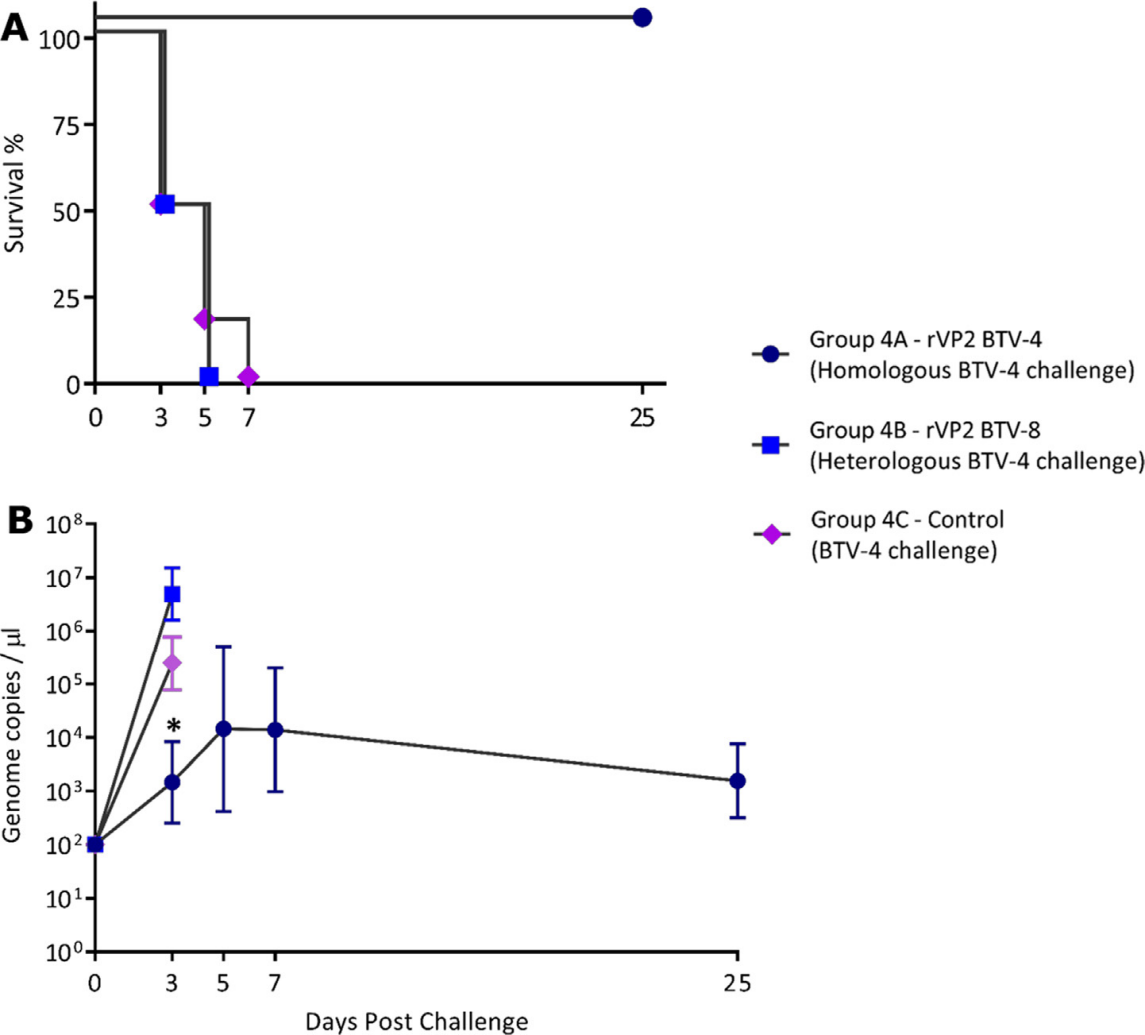
**Survival and mean genome copy numbers/µL for prime/boost vaccinated mice challenged with BTV-4.** Two groups of 6 IFNAR ^−/−^ mice received a prime/boost vaccination 14 days apart with rVP2 of either BTV-4 (group 4A) or BTV-8 (group 4B) prior to challenge with BTV-4, 14 days later. The mock vaccinated negative control group 4C was also challenged with BTV-4. Panel A: Survival Curves. Animals in the homologous challenge group 4A survived for the duration of the challenge study. Three of the mice in the heterologous challenge group 4B had died by day 3 pc, and the three remaining mice had all died by day 5 pc. In the unvaccinated challenge group 4C, three of the mice had died by day 3 pc, two more had died by day 5 pc and the remaining mouse had died by day 7 pc. Panel B: Mean genome copy/µlL of blood from the surviving mice at days 3, 5, 7 and 25 pc. At day 3 pc, viraemia was significanty lower in animals in group 4A compared to those in the control group, 4C (*P = 0.028).

All mice vaccinated with rVP2 BTV-4 survived the homologous serotype challenge with BTV-4 (Fig. 2A), although they did become infected developing levels of viraemia that peaked on days 5 to 7 pc, which then declined by day 25 pc In contrast, three of the mice in both groups 4B (vaccinated with rVP2 of BTV-8) and group 4C (mock vaccinated with PBS) had died by day 3 pc. The three remaining animals in both the heterologous rVP2 and mock vaccinated control groups (groups 4B and 4C), had all died by day 5 pc, except for one animal in the mock vaccinated control group that died by day 7 pc. The mean BTV-4 viraemia on day 3 pc, in group 4A mice vaccinated with the homologous rVP2, was significantly lower (1.89 × 10^3^ genome copies/µL (*P* = 0.028) than in the 3 surviving mice in the mock vaccinated control group 4C (at 2.38 × 10^5^ genome copies/µL), which was also lower than in the three surviving mice in group 4B previously vaccinated with rVP2 of the heterologous serotype BTV-8 (4.28 × 10^6^ genome copies/µL) (Fig. 2B, Table S3).

**Table S3:** 

**Fig. 3:**
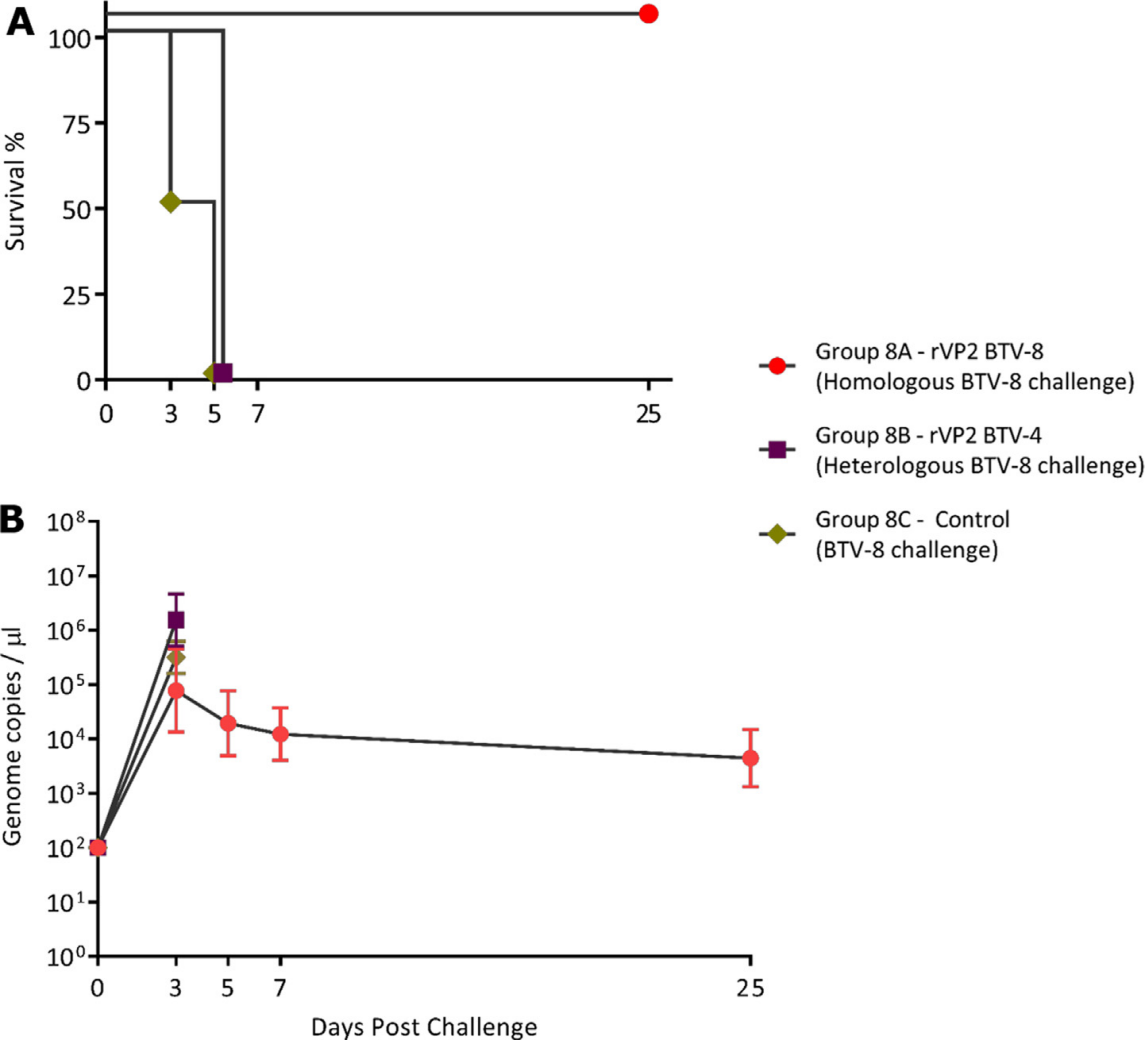
**Survival and mean genome copy numbers/µL for prime/boost vaccinated mice challenged with BTV-8.** Two groups of 6 IFNAR ^−/−^ mice received a prime/boost vaccination 14 days apart, with rVP2 of either BTV-8 (group 8A) or BTV-4 (group 8B) prior to challenge with BTV-8, 14 days later. The mock vaccinated negative control group 8C was also challenged with BTV-8. Panel A: Survival Curves. Animals in group 8A survived for the duration of the challenge study. Mice in group 8B had all died by day 5 pc. In group 8C, three of the mice had died by day 3 pc, the remaining three mice had all died by day 5 pc. Panel B: Mean BTV genome copy/µL of blood from the surviving mice at days 3, 5, 7 and 25 pc.

Group 8A mice had generated a low level of antibodies to rVP2 of BTV-8 by day 0 pc, as detected by both SNT (Table 2) and ELISA (Table 3). These antibodies did not detect rVP2 of BTV-4 in either assay. The group 8A mice challenged with BTV-8 all survived to the end of the experiment on day 25 pc (Fig. 3A). Group 8A mice developed peak viraemia on days 3 to 7 pc (2.38 × 10^4^ to 2.56 × 10^6^ genome copies/µL by day 3pc) (Fig. 3B, Table S4), higher than the BTV-4 viraemia in the equivalent BTV-4 vaccinated/challenge group 4A (which varied from undetectable to 2.17 × 10^4^ BTV-4 genome copies/µL on day 3 pc) (Fig. 2B, Table S4).

**Table 2:** Mean neutralisation titres of mouse-antisera post-vaccination.

Group	nAb titres by SNT (Log_10_) (28 dpv)	
	BTV-4	BTV-8
**Group 4A:** rVP2 BTV-4 prime/boost vaccinated	2.96	NT
**Group 4B:** rVP2 BTV-8 prime/boost vaccinated	<2	NT
**Group 4C:** Control (pooled sera)	<2	NT
**Group 8A:** rVP2 BTV-8 Prime/boost vaccinated	NT	2.16
**Group 8B:** rVP2 BTV-4 prime/boost vaccinated	NT	<2
**Group 8C: Control** **(pooled sera)**	NT	<2

NT = Not tested due to limited volumes. The antisera from mice in groups 4A, 4B, 8A and 8B were tested individually for neutralising antibodies against the relevant, homologous or heterologous challenge strain, prior to challenge (Table S1). Antisera from control groups 4C and 8C were pooled prior to testing. The limit of detection was a neutralisation titre of 2 (Log_10_). No neutralising antibodies were detected in pooled or individual mouse sera at day 0 (pre-vaccination).

**Table 3:** Mean ELISA OD values for mice prime/boost vaccinated with rVP2 of BTV-4 or BTV-8.

Group *(pooled sera)*	Normalised OD ±1 SD			
	Day 0 pv.		Day 28 pv.	
	rVP2 BTV-4	rVP2 BTV-8	rVP2 BTV-4	rVP2 BTV-8
**Group 4A:** rVP2 BTV-4 prime/boost vaccinated	0.07	0.01	0.40	0.11
**Group 4B:** rVP2 BTV-8 prime/boost vaccinated	0.13	0.05	0.07*	0.69*
**Group 4C:** PBS vaccinated control (pooled sera)	0.03 ± 0.01**	0.02 ± 0.01	0.03 ± 0.06	0.16 ± 0.32
**Group 8A:** rVP2 BTV-8 prime/boost vaccinated	0.14	0.09	0.08	0.63
**Group 8B:** rVP2 BTV-4 prime/boost vaccinated	0.14	0.01	0.52	0.07
**Group 8C:** PBS vaccinated control (pooled sera)	0.06 ± 0.05	0.02 ± 0.02	0.00 ± 0.04	0.01 ± 0.14

Data for individual animals in groups 4A, 4B, 8A and 8B are given in Table S2. * Sufficient serum for SNT was only obtained for three of the mice in group 4B. **±1 SD.

**Table S4:** 

The mean level of BTV-8 viraemia in the homologous challenge group 8A, was not significantly different from that detected in the three surviving mice in the mock vaccinated control group 8C at day 3 pc (*P* = 0.121), suggesting that in this study the level of early viraemia is not a good predictor for the severity of clinical outcomes after challenge (Fig. 3B). Mice vaccinated with rVP2 of BTV-4 (group 8B) and mock vaccinated control mice (group 8C) challenged with BTV-8 had all died by day 5 pc confirming the serotype-specific nature of the protective response (Table S5). However, as already observed with the BTV-4 rVP2 vaccinated mice (Table S4), the level of viraemia in the three surviving mock vaccinated mice in group 8C, on day 3 pc (1.71 × 10^5^ to 6.56 × 10^5^ genome copies/µL – mean of 2.73 × 10^5^) (Table S3), was lower than in the heterologous rVP2 BTV-4 vaccinated mice after challenge with BTV-8 (group 8B) at 2.65 × 10^5^ to 6.22 × 10^6^ genome copies/µL – mean of 1.91 × 10^6^ (Table S4). This suggests that the immune response to rVP2 may have increased early replication and the level of viraemia caused by the heterologous BTV serotype, for both BTV-4 and BTV-8.

**Table S5:** 

Virus isolation attempted from EDTA blood samples collected at day 25 pc for groups 4A and 8A were unsuccessful using BSR and KC cell lines (data not shown).

### 3.4 Challenge study using a single dose rVP2 BTV-8 vaccination

**Fig. 4:**
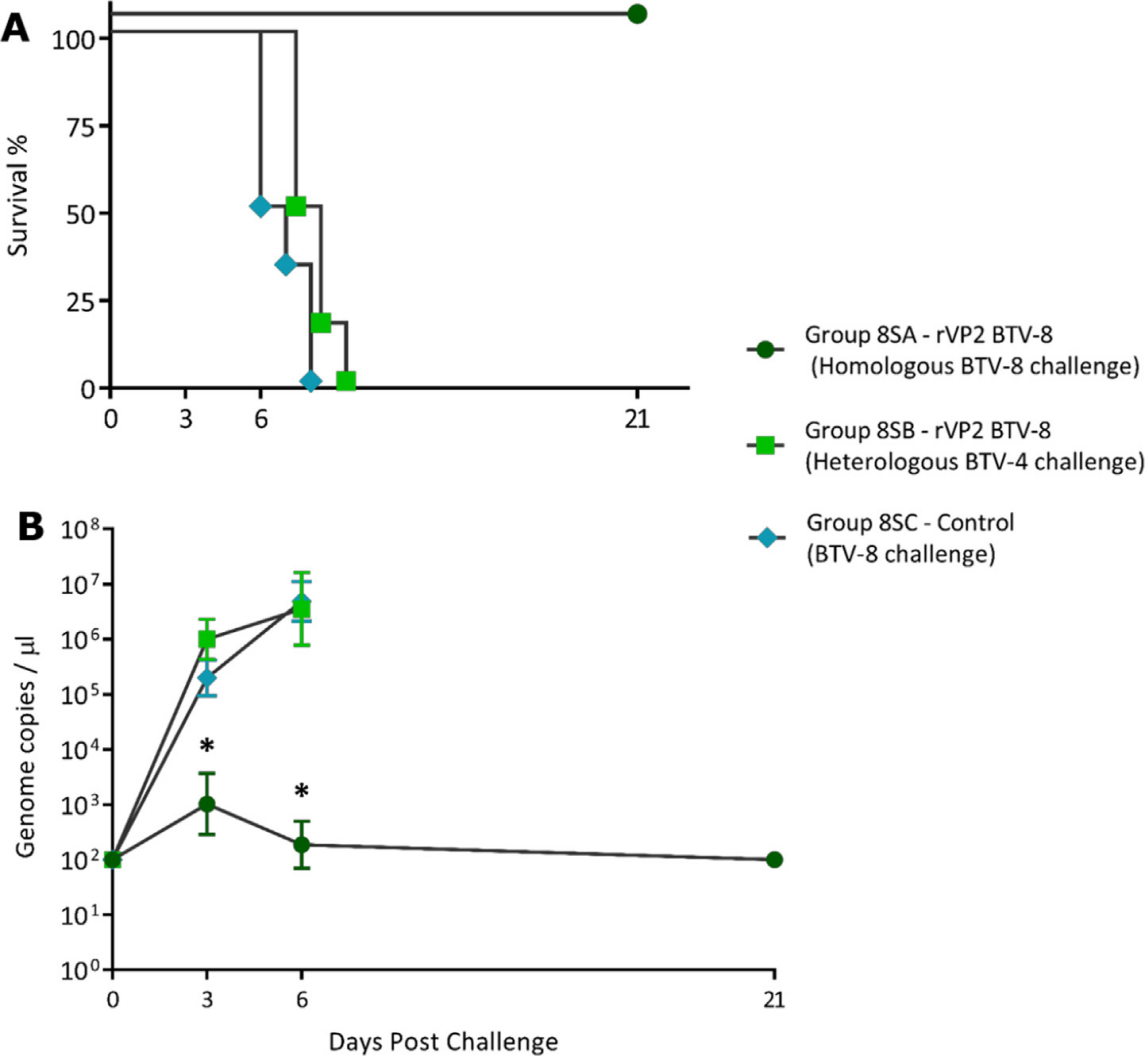
**Survival and mean genome copy numbers/µL in mice after single dose vaccination with rVP2 of BTV-8.** Two groups of 6 IFNAR ^−/−^ mice received a single vaccination with rVP2 of BTV-8 prior to challenge 14 days later. Group 8SA mice were challenged with the homologous BTV-8, while group 8SB mice were challenged with the heterologous BTV-4. The PBS vaccinated control group 8SC was also challenged with BTV-8. Panel A: Survival Curves. Mice in the homologous challenge group 8SA survived for the duration of the challenge study. Mice in the PBS vaccinated control group 8SC had all died by day 8 pc, while those in the heterologous (BTV-4) challenge group 8SB had all died by day 9 pc. Panel B: Mean BTV genome copy/µL of blood from surviving mice at days 3, 6 and 21 pc. At days 3 and 6 pc, viraemia was significantly lower in animals in group 8SA compared to animals in group 8SB and 8SC (*P = <0.05).

A single dose vaccination study was also carried out in IFNAR ^−/−^ mice, using rVP2 of BTV-8, to see if a single shot vaccination would confer protection. Fourteen days post-vaccination the mice were challenged with either BTV-8 or BTV-4 (groups 8SA and 8SB respectively). The PBS vaccinated control group 8SC was also challenged with BTV-8. All of the vaccinated mice in group 8SA, survived for the duration of the study (21 dpc). No cross-protection was detected in mice receiving the BTV-4 heterologous challenge, which had all died by day 8 pc (Fig. 4A).

All of the challenged mice developed viraemia, with a higher level of genome copies/µL detected in group 8SB and 8SC (6.64 × 10^5^ to 2.22 × 10^5^ genome copies/µL, respectively) compared to group 8SA (1.37 × 10^3^ genome copies/µL) by day 3 pc (*P* = 0.05). Peak viraemia was reached by day 6 pc for both the heterologous vaccinated and PBS vaccinated control animals (groups 8SB and 8SC – mean of 6.0 × 10^6^ to 4.79 × 10^6^ genome copies/µL, respectively). However, mice in the homologous group 8SA developed significantly less viraemia compared to mice in groups 8SB and 8SC (*P* = 0.003). Viraemia in the homologous challenge mice dropped after day 3 pc and no RNA was detected in any of these animals by the end of the study (Fig. 4B, Table S5). Unfortunately, insufficient blood was recovered from individual animals in this experiment to evaluate the production of either neutralising or non-neutralising VP2-specific antibodies.

## 4 Discussion

Recombinant VP2s of BTV-4 and BTV-8 were successfully and abundantly expressed as soluble proteins with His-tags in tobacco plants (*N.benthamiana*), then purified by affinity chromatography. These rVP2 proteins raised a serotype-specific and protective immune response in vaccinated IFNAR ^−/−^ mice, in the absence of the other BTV proteins. The vaccinated mice all survived a virulent homologous challenge, and although they still became infected they displayed no clinical signs and maintained body weight. The levels of nAbs that were detected against the homologous BTV serotype, after prime/boost vaccinations with rVP2, are considered likely to have played an important role in the protection observed, although this study does not directly differentiate the roles and importance of neutralising and non-neutralising antibodies, or any potential T-cell mediated response in protection.

Although BTV RNA was detected by real-time RT-qPCR in the surviving homologous challenge groups on day 25 post-challenge, attempts to re-isolate the virus were unsuccessful. This may reflect the haemagglutination activity known to be associated with BTV particles, binding them to circulating erythrocytes even in the presence of nAbs, rather than a significant level of persistent infectivity [3], [35], [36].

Previous studies in IFNAR ^−/−^ mice vaccinated with VP2 purified from intact BTV virions or expressed by recombinant baculovirus, have shown partial protection against virulent challenge with homologous serotypes [37], [38]. Prime/boost vaccination strategies using rDNA/rMVA protocols, expressing a cocktail of VP2 and VP5 from BTV-4, were also partially protective [25], [39]. A similar study design found that IFNAR ^−/−^ mice vaccinated using a rMVA/rDNA or rMVA/rMVA prime/boost strategy for VP2 of BTV-8 alone, were fully protected post-challenge with the homologous virus serotype [29].

Cross-serotype neutralising antibody and protective immune responses have been reported after sequential vaccination/infection with MLV from two different BTV serotypes, which were significantly enhanced following challenge with a third heterotypic serotype (44). The production of cross-reactive nAbs, even at low titres, could prime an enhanced secondary immune response to infection by a heterologous strain, potentially resulting in faster nAb proliferation and a more protective cross-reactive response.

The development of cross-reactive nAbs is thought to reflect the presence of a proportion of the neutralising epitopes on VP2, that are conserved or shared between different BTV serotypes [40]. Earlier studies have reported low-level or one-way serological cross-reactions between different BTV serotypes in cross neutralisation and cross-protection studies, that reflect the phylogenetic relationships observed between their VP2 proteins [41], [42]. However in the study described here, mice vaccinated with rVP2 alone were unprotected against a virulent heterologous-serotype challenge, indicating the absence of a cross-protective immune response between BTV-4 and BTV-8 (which are distantly related VP2 proteins [42]).

After heterologous challenge with either BTV-4 or BTV-8, the surviving single shot and prime/boost rVP2 immunised mice developed a higher mean viraemia on day 3, than in the unvaccinated control mice. Although not conclusive, these results suggest that immunisation with rVP2 could have increased the early replication and viraemia caused by the heterologous challenge virus.

A higher BTV-genome copy number was detected in blood samples taken on day 3 post challenge, from mice that received a prime/boost vaccination with rVP2 of BTV-8, followed by a homologous serotype challenge when compared to mice that had received only a single shot with the same protein. Although this may in part reflect experimental variation, the presence of higher levels of non-neutralising antibodies to rVP2 of BTV-8 in the prime/boost vaccinated group, may have enhanced the early infection of cellular components of the immune system [43], leading to a more rapid early rise in viraemia, even though both groups of mice were protected and survived for the duration of the experiment.

Any potential for vaccine-associated enhancement of infection, could have a major impact on the control and epidemiology of BTV, particularly in areas where more than one serotype is circulating, or multivalent (multi-serotype) vaccines are being used.

The antibodies to rVP2 detected by western blot and ELISA (both of which may be non-neutralising), in sera taken from each of the rVP2 prime/boost vaccination groups on day 28 pv (day 0 pre-challenge), and nAbs detected by SNT, are thought likely to play a significant collective role in the serotype-specific protection that was observed. Although the role of non-neutralising antibodies in protection is unclear, it is possible that during the early stages of infection the level of viraemia could be reduced through opsonisation of infected cells. This could result in a reduction in the development of severe clinical signs and/or a reduction of viraemia below levels transmissible to midges, thus also reducing the risk of onward infection [44].

Cell-mediated immune responses (e.g. against the NS1 ‘tubule’ protein) have been shown to be protective against heterologous BTV serotypes [45]. A strong cell-mediated immune response initiated during early infection that influences the protective humoral response, has been observed in studies with different BTV serotypes, resulting in reduced viraemia prior to the development of neutralising antibodies [45], [46], [47], [48]. It is possible that cell-mediated immune responses (against VP2) could play some role in the protection observed after homologous serotype challenge in the current study.

A strong nAb response is highly dependent on accurate epitope display. It is therefore important when using recombinant expressed VP2 to retain a relevant/native protein structural conformation [37], [49], [50], [51]. The soluble nature of the rVP2 proteins of BTV-4 and BTV-8 that were successfully expressed in tobacco plants, suggests that they retain a native conformation. This expression system eliminates the possibility of contamination with other viral, mammalian or insect host proteins, or contamination with infectious BTV that could affect the range of viral-antigens and consequently the range of antibodies and cross-reactions detected. The plant based methods used do not require containment or large scale sterile facilities for protein production, are readily scalable and very rapid, taking approximately two weeks from construct design to the production of the purified protein, further reducing overall costs [30].

The results described here indicate potential for use of recombinant expressed VP2s as sub-unit vaccine components. The immune response to plant-expressed rVP2 from a wider range of different BTV serotypes (including BTV-4 and BTV-8) is currently being investigated in both mice and sheep. This includes an analysis of the roles of neutralising and non-neutralising antibodies in protection, after both single-shot and prime-boost immunisation and the extent of immune cross-reactivity after sequential or simultaneous vaccination with different rVP2s, before and after challenge with different serotypes. The results obtained will be compared to the known phylogenetic relationships of the VP2 proteins of these viruses [51] and may help in development of efficacious multi-serotype, DIVA compatible vaccines and vaccination strategies, that pose no risk of reassortment, reversion to virulence or onward transmission.
