# Supplementary material for: Bluetongue virus outer-capsid protein VP2 expressed in Nicotiana benthamiana raises neutralising antibodies and a protective immune response in IFNAR −/− mice
Source: Vaccine X. 2019 Jun 22;2:100026. doi: 10.1016/j.jvacx.2019.100026 (PMC6668234; doi:10.1016/j.jvacx.2019.100026)
Supplement: Supplementary file 1 [file mmc3.docx]

**
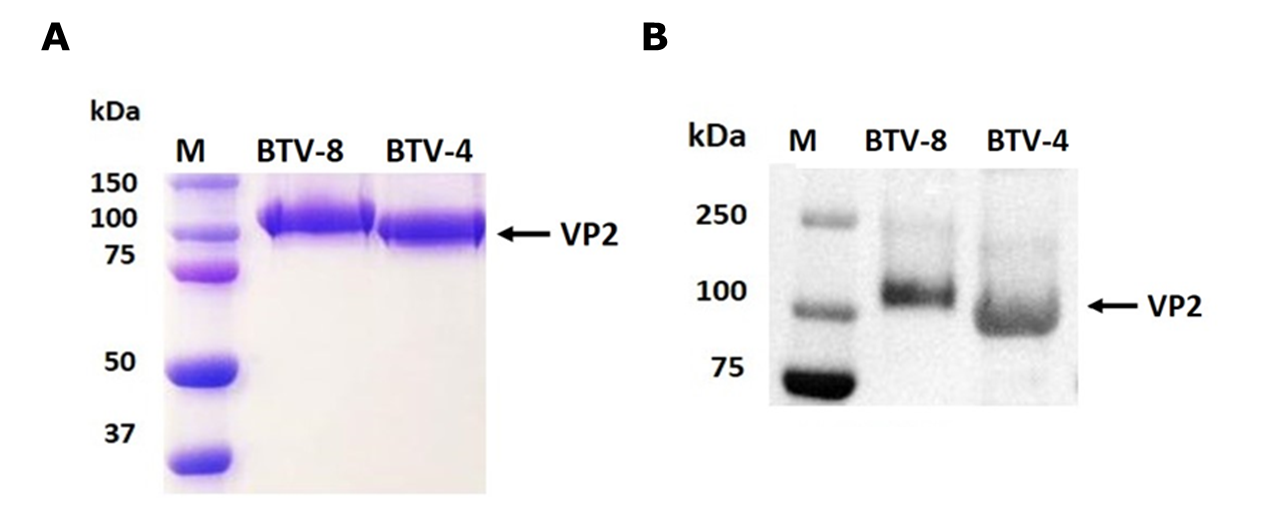
**

**Figure S1.** **Recombinant expression of BTV-4 and BTV-8 VP2 in *N. benthamiana*.**

Plant expressed BTV-rVP2 proteins were purified using IMAC. The soluble His-tagged VP2 proteins were analysed by (A) SDS-PAGE and (B) western blot (using a primary antibody against Penta-His). A major band representative of VP2 monomers was visible in both cases at ~110 kDa.
