# Supplementary material for: Bluetongue virus outer-capsid protein VP2 expressed in Nicotiana benthamiana raises neutralising antibodies and a protective immune response in IFNAR −/− mice
Source: Vaccine X. 2019 Jun 22;2:100026. doi: 10.1016/j.jvacx.2019.100026 (PMC6668234; doi:10.1016/j.jvacx.2019.100026)
Supplement: Supplementary file 2 [file mmc1.docx]

**Table S1: Neutralisation titres of individual prime boost mouse antisera pre-challenge**

| ***Group*** | **Individual mouse**  **number** | **nAb titres by SNT (Log_10_) on Day 28 (pre-challenge)** | |
| --- | --- | --- | --- |
|  |  | BTV-4 | BTV-8 |
| **Group 4A:**  rVP2 BTV-4  prime/boost vaccinated | 4A-1 | 3.08 | NT |
|  | 4A-2 | 3.20 |  |
|  | 4A-3 | 2.90 |  |
|  | 4A-4 | 3.08 |  |
|  | 4A-5 | 2.60 |  |
|  | 4A-6 | 2.90 |  |
| **Group 4B:**  rVP2 BTV-8  prime/boost vaccinated | 4B-1 | <2  <2  <2  <2  <2  <2 |  |
|  | 4B-2 |  |  |
|  | 4B-3 |  | NT |
|  | 4B-4 |  |  |
|  | 4B-5 |  |  |
|  | 4B-6 |  |  |
| **Group 4C:**  Control | 4C-1 to 4C-6 (pooled sera) | No nAbs detected | NT |
| **Group 8A:**  rVP2 BTV-8  prime/boost vaccinated | 8A-1 | NT | 2 |
|  | 8A-2 |  | 2 |
|  | 8A-3 |  | 2 |
|  | 8A-4 |  | 2 |
|  | 8A-5 |  | 2.9 |
|  | 8A-6 |  | 2.08 |
| **Group 8B:**  rVP2 BTV-4  prime/boost vaccinated | 8B-1 |  | <2  <2  <2  <2  <2  <2 |
|  | 8B-2 |  |  |
|  | 8B-3 | NT |  |
|  | 8B-4 |  |  |
|  | 8B-5 |  |  |
|  | 8B-6 |  |  |
| **Group 8C:**  Control | 8C-1 to 8C-6 (pooled sera) | NT | No nAbs detected |

Antisera from mice in each group were tested for neutralising antibodies against the relevant, homologous or heterologous challenge strain, prior to challenge.

No neutralising antibodies were detected in any of the pooled or individual mouse sera at day 0 (pre-vaccination)

NT = Not tested
