# Supplementary material for: Bluetongue virus outer-capsid protein VP2 expressed in Nicotiana benthamiana raises neutralising antibodies and a protective immune response in IFNAR −/− mice
Source: Vaccine X. 2019 Jun 22;2:100026. doi: 10.1016/j.jvacx.2019.100026 (PMC6668234; doi:10.1016/j.jvacx.2019.100026)
Supplement: Supplementary file 3 [file mmc2.docx]

**Table S2: Detection of antibodies to rVP2 of BTV-4 and BTV-8 in sera from individual prime‑boost vaccinated mice, by ELISA**

| ***Group*** | ***Individual Mouse number*** | ***Normalised OD ±1 SD*** | | | |
| --- | --- | --- | --- | --- | --- |
|  |  | **Day 0** | | **Day 28** | |
|  |  | rVP2 BTV-4 | rVP2 BTV-8 | rVP2 BTV-4 | rVP2 BTV-8 |
| **Group 4A:**  rVP2 of BTV-4  prime/boost vaccinated | 4A-1 | 0.09 ±0.00 | 0.00 ±0.00 | 0.19 ±0.24 | 0.08 ±0.00 |
|  | 4A-2 | 0.04 ±0.30 | 0.00 ±0.00 | 0.00 ±0.04 | 0.02 ±0.06 |
|  | 4A-3 | 0.13 ±0.70 | 0.07 ±0.05 | 0.48 ±0.15 | 0.17 ±0.02 |
|  | 4A-4 | 0.06 ±0.00 | 0.00 ±0.05 | 0.87 ±0.17 | 0.17 ±0.23 |
|  | 4A-5 | 0.08 ±0.00 | 0.00 ±0.00 | 0.31 ±0.02 | 0.09 ±0.00 |
|  | 4A-6 | 0.02 ±0.02 | 0.00 ±0.00 | 0.55 ±0.09 | 0.10 ±0.03 |
| **Group 4B:**  rVP2 BTV-8  prime/boost vaccinated | 4B-1 | 0.09 ±0.30 | 0.00 ±0.05 | *No data** | *No data** |
|  | 4B-2 | 0.17 ± 0.13 | 0.10 ±0.08 | *No data** | *No data** |
|  | 4B-3 | 0.07 ±0.03 | 0.07 ±0.08 | *No data** | *No data** |
|  | 4B-4 | 0.32 ±0.05 | 0.07 ±0.08 | 0.00 ±0.00 | 0.62 ±0.10 |
|  | 4B-5 | 0.08 ±0.02 | 0.01 ±0.01 | 0.09 ±0.02 | 0.77 ±0.00 |
|  | 4B-6 | 0.02 ±0.02 | 0.02 ±0.02 | 0.11±0.0 | 0.65 ±0.00 |
| **Group 4C:**  Control | Pooled sera | 0.03 ±0.01 | 0.02 ±0.01 | 0.03 ±0.06 | 0.16 ±0.32 |
| **Group 8A:**  rVP2 BTV-8  prime/ boost vaccinated | 8A1 | 0.18 ±0.04 | 0.06 ±0.06 | 0.03 ±0.11 | 0.35 ±0.08 |
|  | 8A2 | 0.02 ±0.06 | 0.02 ±0.00 | 0.04 ±0.02 | 0.43 ±0.00 |
|  | 8A3 | 0.36 ±0.06 | 0.27 ±0.07 | 0.08 ±0.03 | 0.60 ±0.32 |
|  | 8A4 | 0.17 ±0.00 | 0.05 ±0.00 | 0.14 ±0.04 | 0.83 ±0.17 |
|  | 8A5 | 0.02 ±0.03 | 0.06 ±0.00 | 0.09 ±0.00 | 0.92 ±0.09 |
|  | 8A6 | 0.08 ±0.10 | 0.07 ±0.00 | 0.10 ±0.03 | 0.63 ±0.06 |
| **Group 8B:**  rVP2 BTV-4  prime/ boost vaccinated | 8B-1 | 0.14 ±0.13 | 0.00 ±0.02 | 0.49 ±0.17 | 0.03 ±0.02 |
|  | 8B-2 | 0.08 ±0.02 | 0.02 ±0.01 | 0.78 ±0.47 | 0.13 ±0.11 |
|  | 8b-3 | 0.15 ±0.00 | 0.02 ±0.00 | 0.48 ±0.23 | 0.11 ±0.03 |
|  | 8B-4 | 0.06 ±0.10 | 0.00 ±0.00 | 0.56 ±0.00 | 0.00 ±0.01 |
|  | 8B-5 | 0.19 ±0.03 | 0.02 ±0.04 | 0.45 ±0.17 | 0.07 ±0.07 |
|  | 8B-6 | 0.19 ±0.08 | 0.02 ±0.01 | 0.34 ±0.43 | 0.08 ±0.03 |
| **Group 8C:**  Control | Pooled sera | 0.06 ±0.05 | 0.02 ±0.02 | 0.00 ±0.04 | 0.01 ±0.14 |

***** Insufficient antisera were collected from three of the mice in group 4-B on day 28 dpv for measurement of VP2 specific antibodies by ELISA.
