# Supplementary material for: Bluetongue virus outer-capsid protein VP2 expressed in Nicotiana benthamiana raises neutralising antibodies and a protective immune response in IFNAR −/− mice
Source: Vaccine X. 2019 Jun 22;2:100026. doi: 10.1016/j.jvacx.2019.100026 (PMC6668234; doi:10.1016/j.jvacx.2019.100026)
Supplement: Supplementary file 4 [file mmc4.docx]

**Table S3: BTV genome copy/µL, determined by real-time RT-qPCR, for prime/boost vaccinated IFNAR ^-/-^ mice BTV‑4 challenge groups**

| **Groups**  **(n=6)** | **Mouse**  **number** | **3 days pc.** | | **5 days pc.** | | **7 days pc.** | | **25 days pc.** | |
| --- | --- | --- | --- | --- | --- | --- | --- | --- | --- |
|  |  | ***C_T_*** | **Copy / µL** | ***C_T_*** | **Copy / µL** | ***C_T_*** | **Copy / µL** | ***C_T_*** | **Copy / µL** |
| **Group 4A:**  rVP2 BTV-4 Vaccinated  -  Homologous BTV-4 challenge | 4A-1 | 36.09 | 3.12 x 10^3^ | 35.58 | 3.91 x 10^3^ | 31.92 | 2.22 x 10^4^ | 38.66 | 1.04 x 10^3^ |
|  | 4A-2 | No *C*_T_ | <1 x 10^2^ | No *C*_T_ | <1 x 10^2^ | 33.93 | 8.36 x 10^3^ | 35.44 | 4.17 x 10^3^ |
|  | 4A-3 | 38.76 | 9.97 x 10^2^ | 28.10 | 1.70 x 10^5^ | 39.03 | 8.92 x 10^2^ | No *C*_T_ | <1 x 10^2^ |
|  | 4A-4 | 31.96 | 2.17 x 10^4^ | 24.26 | 1.78 x 10^6^ | 24.88 | 1.19 x 10^6^ | 34.75 | 5.71 x 10^3^ |
|  | 4A-5 | 38.15 | 1.28 x 10^3^ | 38.01 | 1.36 x 10^3^ | 31.09 | 3.38 x 10^4^ | 34.49 | 6.43 x 10^3^ |
|  | 4A-6 | 38.48 | 1.12 x 10^3^ | 29.97 | 6.07 x 10^4^ | 38.61 | 1.06 x 10^3^ | 39.01 | 9.00 x 10^2^ |
| **Mean values |  | 37.24 | 1.89 x 10^3^ | 32.65 | 1.54 x 10^4^ | 33.24 | 1.16 x 10^4^ | 37.23 | 2.04 x 10^3^ |
| **Group 4B:**  rVP2 BTV-8 Vaccinated  -  Heterologous  BTV-4 challenge | 4B-1 | D | - | - | - |  |  |  |  |
|  | 4B-2 | D | - | - | - |  |  |  |  |
|  | 4B-3 | D | - | - | - |  |  |  |  |
|  | 4B-4 | 24.15 | 1.91 x 10^6^ | D | - |  |  |  |  |
|  | 4B-5 | 23.16 | 3.73 x 10^6^ | D | - |  |  |  |  |
|  | 4B-6 | 21.07 | 1.69 x 10^7^ | D | - |  |  |  |  |
| **Mean values |  | 22.79 | 4.28 x 10^6^ |  |  |  |  |  |  |
| **Group 4C:**  PBS vaccinated Control  -  BTV-4 Challenge | 4C-1 | D | - | - | - |  |  |  |  |
|  | 4C-2 | 25.98 | 5.94 x 10^5^ | D | - |  |  |  |  |
|  | 4C-3 | D | - | - | - |  |  |  |  |
|  | 4C-4 | D | - | - | - |  |  |  |  |
|  | 4C-5 | 29.80 | 6.64 x 10^4^ | 23.62 | 2.72 x 10^6^ | D | - |  |  |
|  | 4C-6 | 26.74 | 3.75 x 10^5^ | D | - |  |  |  |  |
| **Mean values |  | 27.51 | 2.38 x 10^5^ | 23.62 | 2.72 x 10^6^ |  |  |  |  |

*D denotes animal death.

** Mean *C*_T_ value for the surviving animals in each group was used to calculate mean genome copy number / µL of blood.

RNA extracted from blood samples were tested using a Seg-10 real-time RT-qPCR assay. No BTV RNA was detected in blood samples taken from animals on day 28 post vaccination / day 0 pre-challenge.
