# Supplementary material for: Bluetongue virus outer-capsid protein VP2 expressed in Nicotiana benthamiana raises neutralising antibodies and a protective immune response in IFNAR −/− mice
Source: Vaccine X. 2019 Jun 22;2:100026. doi: 10.1016/j.jvacx.2019.100026 (PMC6668234; doi:10.1016/j.jvacx.2019.100026)
Supplement: Supplementary file 5 [file mmc5.docx]

**Table S4: BTV genome copy/µL of blood, determined by real-time RT-qPCR, for prime/boost vaccinated IFNAR ^-/-^ mice BTV‑8 challenge groups**

| **Group** | **Mouse**  **number** | **3 days pc.** | | **5 days pc.** | | **7 days pc.** | | **25 days pc.** | |
| --- | --- | --- | --- | --- | --- | --- | --- | --- | --- |
|  |  | ***C_T_*** | **Copy / µL** | ***C_T_*** | **Copy / µL** | ***C_T_*** | **Copy / µL** | ***C_T_*** | **Copy / µL** |
| **Group 8A:**  rVP2 BTV-8 Vaccinated  -  Homologous BTV-8  Challenge | 8A-1 | 31.78 | 2.38 x 10^4^ | 38.23 | 1.24 x10^3^ | 34.06 | 7.86 x 10^3^ | 31.72 | 2.45 x 10^4^ |
|  | 8A-2 | 23.71 | 2.56 x 10^6^ | 31.03 | 3.48 x 10^4^ | 36.37 | 2.76 x 10^3^ | 39.31 | 7.96 x 10^2^ |
|  | 8A-3 | 30.12 | 5.60 x 10^4^ | 31.51 | 2.72 x 10^4^ | 31.19 | 3.21 x 10^4^ | 37.05 | 2.05 x 10^3^ |
|  | 8A-4 | 29.90 | 6.30 x 10^4^ | 30.21 | 5.54 x 10^4^ | 31.27 | 3.08 x 10^4^ | 34.05 | 7.90 x 10^3^ |
|  | 8A-5 | 31.47 | 2.78 x 10^4^ | 31.32 | 3.0 x 10^4^ | 35.21 | 4.62 x 10^3^ | 36.20 | 2.97 x 10^3^ |
|  | 8A-6 | 30.91 | 3.70 x 10^4^ | 31.51 | 2.72 x 10^4^ | 31.04 | 3.46 x 10^4^ | 33.95 | 8.28 x 10^3^ |
| **Mean values |  | 29.65 | 7.2 x10^4^ | 32.30 | 1.83 x 10^4^ | 33.19 | 1.19 x 10^4^ | 35.38 | 4.28 x 10^3^ |
| **Group 8B:**  rVP2 BTV-4 Vaccinated  -  Heterologous  BTV-8  Challenge | 8B-1 | 24.35 | 1.67 x 10^6^ | D | - |  |  |  |  |
|  | 8B-2 | 23.12 | 3.83 x 10^6^ | D | - |  |  |  |  |
|  | 8B-3 | 24.97 | 1.12 x 10^6^ | D | - |  |  |  |  |
|  | 8B-4 | 27.33 | 2.65 x 10^5^ | D | - |  |  |  |  |
|  | 8B-5 | 22.43 | 6.22 x 10^6^ | D | - |  |  |  |  |
|  | 8B-6 | 24.89 | 1.18 x 10^6^ | D | - |  |  |  |  |
| **Mean values |  | 24.52 | 1.19 x 10^6^ |  |  |  |  |  |  |
| **Group 8C:**  PBS vaccinated Control  -  BTV-8 Challenge | 8C-1 | 27.23 | 1 x 10^9^ | D | - |  |  |  |  |
|  | 8C-2 | 25.82 | 1 x 10^9^ | D | - |  |  |  |  |
|  | 8C-3 | 28.09 | 1 x 10^7^ | D | - |  |  |  |  |
|  | 8C-4 | D | - | D | - |  |  |  |  |
|  | 8C-5 | D | - | D | - |  |  |  |  |
|  | 8C-6 | D | - | D | - |  |  |  |  |
| **Mean values |  | 27.05 | 2.73 x 10^5^ |  |  |  |  |  |  |

*D denotes animal death. RNA extracted from blood samples were tested using a Seg-10 RT-qPCR

** Mean *C*_T_ value for the surviving animals in each group was used to calculate mean genome copy number / µL of blood.

RNA extracted from blood samples were tested using a Seg-10 real-time RT-qPCR assay. No BTV RNA was detected in blood samples taken from animals on day 28 post vaccination / day 0 pre-challenge.
