# Supplementary material for: Bluetongue virus outer-capsid protein VP2 expressed in Nicotiana benthamiana raises neutralising antibodies and a protective immune response in IFNAR −/− mice
Source: Vaccine X. 2019 Jun 22;2:100026. doi: 10.1016/j.jvacx.2019.100026 (PMC6668234; doi:10.1016/j.jvacx.2019.100026)
Supplement: Supplementary file 6 [file mmc6.docx]

**Table S5: BTV genome copy/µL of blood determined by real-time RT-qPCR, in mice vaccinated with a single dose of rVP2 BTV-8 then challenged with BTV-4 or BTV-8**

| **Group** | **Mouse**  **number** | **3 days pc.** | | **6 days pc.** | | **21 days pc.** | |
| --- | --- | --- | --- | --- | --- | --- | --- |
|  |  | ***C_T_*** | **Copy / µL** | ***C_T_*** | **Copy / µL** | ***C_T_*** | **Copy / µL** |
| **Group 8SA:**  rVP2 BTV-8 Vaccinated  -  Homologous BTV-8  Challenge | 8SA-1 | 35.40 | 4.24 x 10^3^ | 39.80 | 6.53 x10^2^ | No *C*_T_ | <1 x 10^2^ |
|  | 8SA-2 | 37.20 | 1.92 x10^3^ | No *C*_T_ | <1 x 10^2^ | No *C*_T_ | <1 x 10^2^ |
|  | 8SA-3 | 39.20 | 8.32 x10^2^ | No *C*_T_ | <1 x 10^2^ | No *C*_T_ | <1 x 10^2^ |
|  | 8SA-4 | No *C*_T_ | <1 x 10^2^ | No *C*_T_ | <1 x 10^2^ | No *C*_T_ | <1 x 10^2^ |
|  | 8SA-5 | 38.70 | 1.02 x 10^3^ | 39.70 | 6.80 x 10^2^ | No *C*_T_ | <1 x 10^2^ |
|  | 8SA-6 | 37.50 | 1.69 x 10^3^ | No *C*_T_ | <1 x 10^2^ | No *C*_T_ | <1 x 10^2^ |
| **Mean values |  | 38.00 | 1.37 x10^3^ | 39.91 | 6.22 x 10^2^ | No *C*_T_ | <1 x 10^2^ |
| **Group 8SB:**  rVP2 BTV-8 Vaccinated  -  Heterologous  BTV-4  Challenge | 8SB-1 | 27.50 | 2.40 x 10^5^ | 22.50 | 2.4 x 10^5^ |  |  |
|  | 8SB-2 | 23.70 | 2.58 x 10^6^ | 21.40 | 1.32 x 10^7^ |  |  |
|  | 8SB-3 | 24.60 | 1.42 x 10^6^ | 21.20 | 1.53 x 10^7^ |  |  |
|  | 8SB-4 | 28.30 | 1.52 x 10^6^ | 23.90 | 2.25 x 10^6^ |  |  |
|  | 8SB-5 | 24.90 | 1.17 x 10^6^ | 22.70 | 5.13 x 10^6^ |  |  |
|  | 8SB-6 | 25.80 | 6.64 x 10^5^ | 23.20 | 3.62 x 10^6^ |  |  |
| **Mean values |  | 25.80 | 6.64 x 10^5^ | 22.50 | 6.00 x 10^6^ |  |  |
| **Group 8SC:**  PBS vaccinated Control  -  BTV-8 Challenge | 8SC-1 | 28.30 | 1.52 x 10^5^ | 23.80 | 2.41 x 10^6^ |  |  |
|  | 8SC-2 | 24.50 | 1.52 x 10^6^ | 21.40 | 1.32 x 10^7^ |  |  |
|  | 8SC-3 | 27.60 | 2.26 x 10^5^ | 21.70 | 1.05 x 10^7^ |  |  |
|  | 8SC-4 | 27.20 | 2.86 x 10^5^ | 23.60 | 2.76 x 10^6^ |  |  |
|  | 8SC-5 | 25.90 | 6.24 x 10^5^ | 22.20 | 7.33 x10^6^ |  |  |
|  | 8SC-6 | 29.80 | 6.64 x 10^4^ | 24.10 | 1.97 x 10^6^ |  |  |
| **Mean values |  | 27.21 | 2.22 x 10^5^ | 22.80 | 4.79 x 10^6^ |  |  |

RNA extracted from blood samples was tested using a Seg-10 real-time RT-qPCR assay.

** Mean *C*_T_ value for the animals in each group was used to calculate mean genome copy number / µL of blood.

No BTV RNA was detected in blood samples taken from animals on day 14 post vaccination / day 0 pre-challenge.
